# A systematic review of inflammatory cells and markers in human tendinopathy

**DOI:** 10.1186/s12891-020-3094-y

**Published:** 2020-02-06

**Authors:** George Jomaa, Cheuk-Kin Kwan, Sai-Chuen Fu, Samuel Ka-Kin Ling, Kai-Ming Chan, Patrick Shu-Hang Yung, Christer Rolf

**Affiliations:** 1 Division of Orthopedics and Biotechnology, Department of Clinical Science, intervention and Technology (CLINTEC), Karolinska Institutet, Solna, Sweden; 2 Department of Orthopaedics and Traumatology, Faculty of Medicine, The Chinese University of Hong Kong, Hong Kong, People’s Republic of China; 3 Lui Che Woo Institute of Innovative Medicine, The Chinese University of Hong Kong, Hong Kong, People’s Republic of China

**Keywords:** Tendinopathy, Tendon rupture, Inflammation, Immune system, Inflammatory mediators

## Abstract

**Background:**

This article systematically reviews the current evidence regarding inflammation in Tendinopathy with the aim to increase understanding of a potential common pathophysiology.

**Methods:**

Following the PRISMA statements, the terms: (tendinopathy OR (tendons AND rupture)) AND (inflammation OR (inflammation AND cells) OR immune system OR inflammation mediators OR bacteria) were used. One thousand four hundred thirty-one articles were identified which was screened down to 53.

**Results:**

39/53 studies mentioned inflammatory cells but had contradicting conclusions. Macrophages were the most common cell type and inflammatory markers were detectable in all the articles which measure them.

**Conclusions:**

The included studies show different conclusions, but this heterogeneity is not unexpected since the clinical criteria of ‘tendinopathy’ encompass a huge clinical spectrum.

Different ‘tendinopathy’ conditions may have different pathophysiology, and even the same clinical condition may be at different disease stages during sampling, which can alter the histological and biochemical picture. Control specimen sampling was suboptimal since the healthy areas of the pathological-tendon may actually be sub-clinically diseased, as could the contralateral tendon in the same subject.

Detection of inflammatory cells is most sensitive using immunohistochemistry targeting the cluster of differentiation markers, especially when compared to the conventional haematoxylin and eosin staining methods. The identified inflammatory cell types favour a chronic inflammatory process; which suggests a persistent stimulus. This means NSAID and glucocorticoids may be useful since they suppress inflammation, but it is noted that they may hinder tendon healing and cause long term problems.

This systematic review demonstrates a diversity of data and conclusions in regard to inflammation as part of the pathogenesis of Tendinopathy, ranging from ongoing or chronic inflammation to non-inflammatory degeneration and chronic infection. Whilst various inflammatory markers are present in two thirds of the reviewed articles, the heterogenicity of data and lack of comparable studies means we cannot conclude a common pathophysiology from this systematic review.

## Background

Tendinopathy affect millions of people in both the athletic and general population, causing great socioeconomical impacts [1]. Despite the presence of various modalities of conservative and surgical treatments, more than one third of the patients do not respond and continue to present with persistent pain and disability [2, 3]. Anti-inflammatory treatments including NSAIDs and glucocorticoids form the back-bone of conservative treatments for tendinopathy. However, there is still an ongoing debate on the presence of active inflammation in this chronic disorder. Whether inflammation play an important role in tendinopathy is currently unclear. By reviewing the presence and pattern of inflammation in tendinopathic tendons, the current management strategies can be re-assessed.

There have been an ongoing debate on whether an active inflammation is present in chronic tendinopathy. Tendon healing was proposed to be separated into 3 overlapping steps including inflammation, proliferation, and remodelling. The inflammatory phase typically lasts for no longer than weeks, and the presence of a functional, regulated inflammatory process is crucial in maintaining the integrity of tendon tissues [4, 5].

Previously, the term “tendinitis” have been commonly used to describe the clinical symptom of pain and disability of a tendon [6]. However, as argued by an editorial published in 1998, it was suggested that the usage of the term “tendinitis” should be limited to histological findings, and “tendinopathy” is a more suitable vocabulary to describe pain and deranged function of the tendon in a clinical setting [7]. It was also suggested in this editorial that using “tendinitis” to describe chronic tendon disorders is again inaccurate and misleading since tendinopathic tendons were said to present as a degenerative lesion with an absence of inflammatory cells [7, 8].

Contrarily, inflammatory cells and markers have been reported to be present or increased in tendinopathic tendons in recent studies [9]. It was suggested that defects may occur in the cellular responses regulating the inflammatory process, leading to a poor resolution of inflammation. It was further suggested that chronic inflammation may persist in the injured tendon, leading to possible further damage and ultimately the degenerative changes observed in chronic tendinopathy [10]. A recent systematic review of 5 studies have suggested the presence of inflammatory cells in painful tendinopathy [6]. However, the character of inflammation present in tendinopathic tendons is yet to be identified, and current theories of pathogenesis could not satisfactorily explain the varying presentation of inflammation observed in tendinopathic tendons. It is strongly suspected that more studies could be reviewed to facilitate the discussion of this topic.

The aim of this study is to systematically review whether tendinopathy involves an on-going inflammatory process in terms of the presence of inflammatory cells. Any reported changes in inflammatory markers will also be assessed. We would also take an ambitious step to discuss whether the current anti-inflammatory approach to conservatively manage tendinopathy is appropriate, provide a discussion on why the current management outcomes are sub-optimal, and suggest how we may be able to tackle this clinical problem alternatively. A secondary aim is to identify possible initiators of inflammation, such as trauma, mechanical stresses, inflammatory diseases or infections.

## Methods

Systematic searches were carried out in November 2017 using PubMed, Scopus, Web of Science and Embase. An updated search was conducted in the 4 databases in December 2018. No limits or filters were used. No restrictions were made on language, publication date, and publication status.

The PRISMA statements [11] was used as guidelines in the performance of this systematic review. The keywords in combination with search operants were as follows: (tendinopathy OR (tendons AND rupture)) AND (inflammation OR (inflammation AND cells) OR immune system OR inflammation mediators OR bacteria).

### Eligibility criteria

The inclusion criteria for studies in this systematic review consisted of the following:

Clinical studies investigating the presence of inflammation in tendinopathic tendons such as cross-sectional studies, case-control studies, prospective observational studies, randomized controlled trials. In vitro studies using tendon tissue, or cells derived from tendinopathic tendons were also included. Included studies were restricted to studies with an evidence level of 3 or better. In vitro studies using tissue, or cells that have been treated with cytokines or other agents or modified mechanically were excluded.

Studies with participants of any age presenting with tendinopathy were included. Specimens from spontaneous tendon ruptures were included considering the assumption that only tendinopathic tendons are prone to spontaneous ruptures. Diagnostic criteria of the above diseases include a clinical presentation of chronic pain or loss of function, confirmed with imaging modalities such as magnetic resonance imaging or ultrasound. Studies investigating the presence of inflammatory cells, immune cells, inflammatory markers in tendinopathic tendons, and tendon ruptures were included. Tendinopathy was defined as pain, diffuse or localized swelling, and impaired performance of the tendon. Tendon rupture was defined as a tear, visible with medical imaging, such as MRI or ultrasound, or macroscopically visible. Inflammatory, and immune cells were defined as leukocytes, neutrophils, eosinophils, basophils, mast cells, macrophages, monocytes, T-lymphocytes, B-lymphocytes, NK-cells, and dendritic cells. Inflammation markers were defined as fibroblast growth factors (FGF), platelet-derived growth factor (PDGF), transforming growth factor beta superfamily proteins (TGF-beta superfamily proteins), eicosanoids, COX-1, COX-2, and cytokines. Studies investigating the presence of possible initiators of inflammation, such as bacteria, trauma, mechanical stresses, inflammatory diseases, or other proposed factors were also included.

### Study selection and data collection

**Fig. 1:**
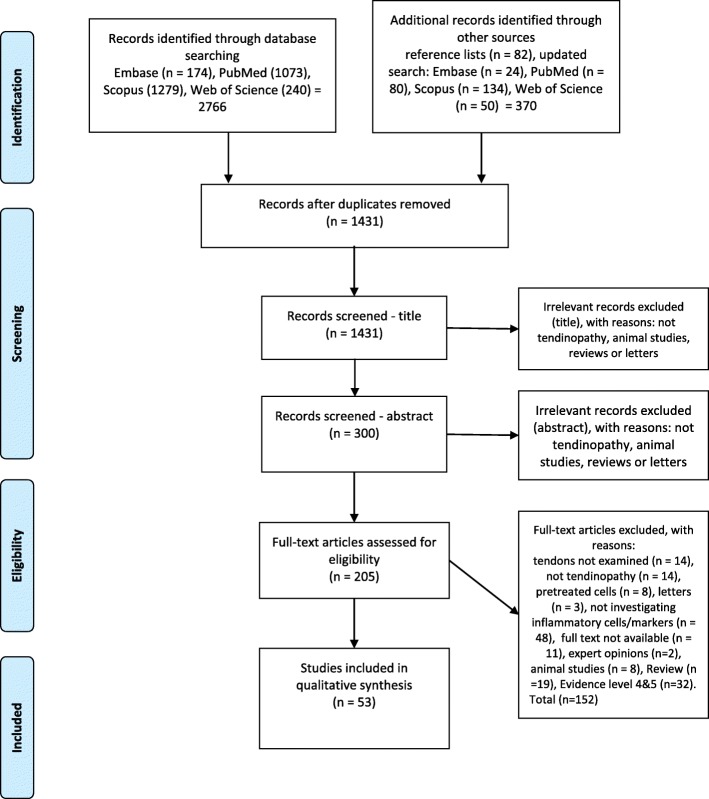
PRISMA (Preferred Reporting Items for Systematic Reviews and Meta-Analyses) flowchart showing results of database search in PubMed, Scopus, Embase and Web of Science

Studies from the search were merged in EndNote, and duplicates were removed. Application of exclusion, and then inclusion criteria were made by screening the titles and then the abstracts. The full texts were then obtained for the identified articles in order for data extraction. A PRISMA-flowchart of the study selection process is shown in Fig. 1. Studies were also identified by screening the reference lists.

### Data collection process & data items

A data extraction table was created. The data extraction for half of the studies was performed by one review author (GJ). The data extraction for the other half of the studies was performed by the second review author (CK). Control of the data extracted were performed in the same way.

### Assessment of study quality and risk of bias in individual studies

Critical Appraisal Skills Programme (CASP) [12] appraisal form was used to assess the quality of included studies. The assessment of study quality was performed in an unblinded standardized manner independently by one reviewer (GJ) and control of the assessment was then assessed by a second reviewer (CK).

### Statistics

Due to the heterogeneity of the studies, e.g. study type, and outcome measures a meta-analysis could not be performed.

## Results

### Studies included

Using the search method mentioned above, 53 studies were included in this review. A total of 2306 tendinopathic tendon specimens were assessed. Tendon specimens were heterogenous in terms of location and presentation of tendinopathy. The details of the included studies will be included in the Table 1.

**Table 1:** Data extraction table

Study	Sample size	Presentation of tendinopathy	Additional factors associated with inflammation^a^	Site	QA	Method (cells)	Inflammatory cell + ^b^	Cell types	Method (markers)	Inflammatory markers
Campbell 2014 [13]	18	Partial tear	Mean number of steroid injections 1.5	RC	8/11	IHC	100%	MP	qPCR	IL-21 receptor protein and mRNA
Cetti 2003 [14]	60	Rupture	High activity level	AT	8/11	IHC	100%	NP	n/a	n/a
Dakin 2015 [15]	32	Pain or partial tear	n/a	RC	9/11	IHC	100%	MP	n/a	n/a
Dakin 2017 [16]	17	Pain or rupture	n/a	AT	7/11	IHC	100%	MP	qPCR, IF	IL-8 mRNA, protein
Hackett 2016 [17]	39	Calcific	n/a	RC	9/11	IHC	100%	MP, TC, MC	n/a	n/a
Klatte-schulz 2018 [18]	26	Pain or ruptures	High activity level	AT	8/11	IHC	88%	MP, NS	qPCR	IL-6, IL-10, IL-33. IL1B, TNFa, TGFB1, COX-2
Kragsnaes 2014 [19]	50	Pain	44% received steroid injection	AT	9/11	IHC	96%	MP, TC, MC, NKC	n/a	n/a
Matthews 2006 [20]	38	Rupture	Mean number of steroid injections 1.8	RC	8/11	IHC	100%	MP, TC, MC	n/a	n/a
Millar 2010 [21]	20	Pain or partial tear	High activity level with mean number of 1.5 steroid injections	RC	8/11	IHC	100%	MP, TC, MC	n/a	n/a
Millar 2012 [22]	15	Pain or partial tear	High activity level with mean number of 1.6 steroid injections	RC	8/11	IHC	100%	MP, TC, MC	IHC, qPCR	IL-6, IL-8 protein and mRNA
Millar 2016 [23]	10	Pain or partial tear	High activity level with mean number of 1.7 steroid injections	RC	7/11	IHC	100%	MP, TC, MC	qPCR	IL-17A mRNA
Mosca 2017 [24]	13	Pain	n/a	RC	9/11	IHC	100%	MP	qPCR	IL-33 protein
Pecina 2010 [25]	34	Pain	n/a	PT	8/11	IHC	100%	NS	n/a	n/a
Schubert 2005 [26]	10	Pain or rupture	40% received steroid injection	AT	6/11	IHC	80%	MP, TC, GC	n/a	n/a
Scott 2008 [27]	22	Pain	n/a	PT	7/11	IHC	23%	MC	n/a	n/a
Thankam 2017 [28]	15	Pain or partial tear	Glenohumeral arthritis observed	LHBT	7/11	IHC	27%	MP, NP	n/a	n/a
Åström 1995 [29]	145	Pain or partial tear	n/a	AT	6/11	H&E	9%	NS	n/a	n/a
Gaida 2012 [30]	23	Pain	n/a	AT	8/11	H&E	0%	/	ELISA	TNFa protein
Gumina 2006 [31]	38	Partial tear	n/a	RC	10/11	H&E	100%	MP, LC	n/a	n/a
Kannus 1991 [32]	891	Rupture	n/a	Various	9/11	H&E	0%	/	n/a	n/a
Khan 1996 [33]	28	Pain	n/a	PT	9/11	H&E	0%	/	n/a	n/a
Lian 2007 [34]	23	Pain	n/a	PT	7/11	H&E	0%	/	n/a	n/a
Ljung 1999 [35]	6	Pain	n/a	ECRB	6/11	H&E	0%	/	n/a	n/a
Longo 2008 [36]	88	Rupture	n/a	RC	8/11	H&E	0%	/	n/a	n/a
Longo 2009 [37]	51	Rupture	n/a	LHBT	8/11	H&E	0%	/	n/a	n/a
Popp 1997 [38]	11	Pain	n/a	PT	5/11	H&E	0%	/	n/a	n/a
Potter 1995 [39]	20	Pain	n/a	ECRB	7/11	H&E	0%	/	n/a	n/a
Rolf 1997 [40]	60	Pain	72% received NSAIDs and 27% received steroid injection	AT	8/11	H&E	0%	/	n/a	n/a
Rolf 2017 [41]	20	Rupture	Sign of infection in 40% (bacteria), 5% received steroid injection 7/20 high activity level	AT	9/11	H&E	50%	MP, TC, MC	n/a	n/a
Shalabi 2002 [42]	15	Pain	n/a	AT	8/11.	H&E	0%	/	n/a	n/a
Singaraju 2008 [43]	6	Pain	n/a	LHBT	6/11	H&E	100%	NS	n/a	n/a
Tillander 2002 [44]	23	Pain, partial tear or rupture	n/a	RC	6/11.	H&E	0%	/	n/a	n/a
Zabrzynski 2017 [45]	35	Pain	n/a	LHBT	9/11	H&E	9%	NS	n/a	n/a
Ackermann 2013 [46]	18	Rupture	n/a	AT	7/11	n/a	n/a	/	MD	IL-12, IL-17, IL-1B, IL-6, IL-8, IL-10 protein
Alfredson 2000 [47]	4	Pain	n/a	ECRB	6/11	n/a	n/a	/	MD	PGE2
Alfredson 2003 [48]	10	Pain	n/a	AT	7/11	n/a	n/a	/	MA	IL 1–6, 10–15 mRNA
Alfredson 2001 [49]	10	Pain	n/a	PT	7/11	H&E	n/a	NS	MD	PGE2
Alfredsson 1999 [50]	5	Pain	n/a	AT	6/11	n/a	n/a	/	MD	PGE2
Chaudhury 2016 [51]	16	Pain	n/a	RC	8/11	n/a	n/a	/	qPCR	IL-8 mRNA
Dean 2015 [52]	9	Pain	100% received steroid injection	RC	7/11	n/a	n/a	/	qPCR	TNF-a, IL-1b mRNA
Fabis 2014 [53]	9	Rupture	n/a	RC	7/11	n/a	n/a	/	qPCR	TNF-a, IL-10 mRNA
Fu 2002 [54]	11	Pain	n/a	PT	7/11	n/a	0%	/	IHC, WB	COX-2, TGF-b protein, PGE2
Gilmer 2015 [55]	62	Pain or partial tear	n/a	LHBT	9/11	H&E	n/a	NS	n/a	n/a
Jelinsky 2011 [56]	23	Pain, tear, or rupture	52% received steroid injection	Various	8/11	n/a	0%	/	qPCR	IL13A2, FGFR1, FGFR2, IL-17D mRNA
Jozsa 1980 [57]	120	Rupture or calcific	n/a	Various	8/11	H&E	n/a	NS	n/a	n/a
Legerlotz 2012 [58]	20	Pain or rupture	n/a	AT PT	7/11	n/a	n/a	/	qPCR	COX-2, IL6, IL6R mRNA
Millar 2015 [59]	17	Pain or partial tear	n/a	RC	8/11	n/a	n/a	/	qPCR	IL-33 mRNA
Pingel 2012 [60]	14	Pain	100% received steroid injection	AT	8/11	n/a	n/a	/	qPCR	COX-1, IL-1R, TGF-B1, bFGF mRNA
Pingel 2013 [61]	27	Pain	100% received steroid injection	AT	8/11	n/a	n/a	/	qPCR	IL-1b, IL-6, IL-10, COX-2, TGF-b, TNF-a mRNA
Robertson 2012 [62]	35	Partial tear or rupture	n/a	RC	8/11	n/a	n/a	/	qPCR	IL-1b, IL-6, TNF-a, COX-2 mRNA
Shindle 2011 [63]	24	Partial tear or Rupture	Joint inflammation	RC	8/11	n/a	n/a	/	qPCR	IL-1b, IL-6, COX-2, TNF-a mRNA
Takeuchi 2001 [64]	7	Calcific	n/a	RC	5/11	IHC	n/a	MP	n/a	n/a
Waugh 2015 [65]	10	Pain	n/a	AT PT	7/11	n/a	n/a	/	MD	IL-1b, IL-2, IL-6,IL-8, IL-10 protein

Table sorted by method used to detect the presence of inflammatory cells

Locations: *AT* Achilles tendon, *PT* patellar tendon, *RC* rotator cuff tendon, *QT* Quadriceps tendon, *LHBT* Long head biceps tendon, *ECRB* extensor carpi radialis brevis tendon, *CF* Common flexor tendon, *CE* Common extensor tendon, various (studies with specimens from more than 3 locations)

Cells: *MP* Macrophages, *MC* Mast-cells, *TC* T-cells, *LC* Lymphocytes, *NP* Neutrophils, *GC* granulocytes, *NKC* NK-cells, *NS* not specified

Detection method: *H&E* Hematoxylin & Eosin, *qPCR* quantitative polymerase chain reaction, *IHC* immunohistochemistry, *IF* immunofluorescence, *MD* Micro-dialysis, *WB* western blot, *EIA* enzyme immunoassay, *ELISA* Enzyme-Linked ImmunoSorbent Assay

^a^ Additional factors associated with inflammation description on patient subgroup and previous treatments

^b^Inflammatory cell + refers to the percentage of specimens showing at least 1 kind of inflammatory cells, regardless of typing

### Methodological quality assessment

The results of the quality assessment are shown in the Table 1. The evidence level of included studies was assessed according to the Oxford Centre of Evidence based Medicine (OCEBM) [66]. All included studies included level 3 evidence (*n* = 53) regarding the presence of inflammatory cells in tendinopathic tendons. 50/53 studies scored 6 or higher out of 11 in CASP. It is notable that included studies have varying objectives, hence the results from quality assessment may not directly reflect how strictly inflammation in tendinopathic specimens were assessed.

### Signs of inflammation in tendinopathic tendons

Among the 53 included studies, 39 studies showed signs of inflammation in tendinopathic tendons, including the presence of inflammatory cells or an increase in inflammatory markers. The expression of inflammation does not seem to be correlated with any obvious confounders including site, presence of rupture, chronicity, or previous treatment of corticosteroid injections.

Twenty-five studies reported the presence of inflammation in the tendon specimens, and 14 suggested the absence of inflammatory cells. By using H&E to assess for inflammatory cells, tendinopathic tendons presenting with inflammatory cells were reported to range from 0 to 100% (mean 16%) between studies. For studies adapting IHC to stain for inflammatory cells, tendinopathic tendons showing inflammatory cells were reported to be 23 to 100% between studies (mean 88%).

For the 25 studies which supported the presence of inflammatory cells, 7 of the studies did not specify which types of cells were observed. The most common cell type was macrophages, found in 16 studies. Other cells include lymphocytes in 2 studies, mast cells in 8 studies, and granulocytes in 3 studies.

Twenty-two studies performed assessment on related inflammatory markers including at least one of the markers mentioned above. All studies indicated increased levels of some inflammatory markers measured. Inflammatory markers reported include IL-1. IL-6, IL-8, IL-10, IL-17, IL-33, COX-1, COX-2, TGF-b, TNF-a, FGF and more. A full list of inflammatory mediators detected can be found in the Table 1.

### Additional factors associated with inflammation

A variety of tendinopathic cases were included from the search, in terms of different locations and presentations.

It is also worth mentioning that data such as previous conservative treatments and activity levels were often non-recorded, or is inconsistent between the patients in the included studies. 19/53 studies included such additional description to the population sub-group that may influence the inflammatory status. In 12 studies, patients have undergone corticosteroid injection. In 5 studies, tendinopathic patients were reported to have experienced high activity level and overuse. In 2 studies, inflammation to the neighbouring structures such as glenohumeral arthritis or joint inflammation were identified as a source of inflammation affecting the tendon. In 1 study, bacteria was identified as a possible source of inflammation. In 1 study, there was a reported use of NSAIDs the recruited tendinopathic patients. Nevertheless, inflammation may be observed in tendinopathic tendons of any size of tear, location, or tendons previously treated with corticosteroid injection.

## Discussion

### Limitations

Heterogenicity of tendinopathic cases is a major limitation of the current review. In this review we included cases with a range of presentations from chronic pain to ruptured cases. Chronicity of the tendon disorder also varied greatly, which when combined may have a great impact on the presentation of inflammation in these specimens included.

Heterogenicity of the detection methods in both the presence of inflammatory cells and inflammatory markers were also significant in this review, making included studies less comparable. However, by comparing the results from different detection methods, we were able to identify a possible explanation in the emergence of the debate on whether chronic inflammation is present in tendinopathic tendons. This will be further discussed in the following section.

Heterogeneity of control specimens is also a limitation of the consistency between studies. In some cases, specimens from macroscopically healthy areas of tendinopathic tendons have been used as controls [60]. This practice is not recommended since it is possible that the whole tendon is affected by tendinopathy [67]. In other studies, the contralateral tendon has been used as control, even though some evidence suggests that unilateral rupture is preceded by bilateral damage [14]. A seemingly healthy tendon could be subclinical and asymptomatic. Tendons from a different anatomical location have also been used as controls [67]. There is a possibility that tendons found on different anatomical localization have a divergent biomechanical construction, due to the fact that the tendons are involved in different movements. Lastly, for several studies, specimens from cadavers have been used as controls. Tendon specimens from this group most likely represents a true healthy tendon. The inconsistency in control groups may have a particularly high impact when comparing inflammatory markers presented in tendinopathic tendons.

The sampling of tendinopathic specimens presenting in different stages was also difficult. Specimens from the debridement of ruptured tendons is common, but these specimens could only represent cases of chronic tendinopathy with an acute insult of tendon rupture. Some studies identified earlier stages of tendinopathy with various methods. There were articles which sampled the macroscopically intact sub-scapularis tendon beside a ruptured supraspinatus [21, 22, 59], and there was another study which defined early pathology as an impinged tendon sampled during acromial decompression [15]. Tendon specimens obtained from the listed protocols mostly represent cases which have likely received and failed other modalities of conservative treatment. Tendinopathy that would respond to conservative treatment also comprise a large part of the total cases. However, tendon specimens were never obtained for these cases due to ethical reasons.

Another limitation is that the demographic data of tendinopathic patients were not reported consistently between studies. For example, it is suspected that the usage of NSAIDs or corticosteroid injections could have a direct effect on the presentation of local inflammation within the tendon. Activity level also have potential effects on the presentation. However, these information is often neglected.

These concerns however reflect the current standpoint in this area of research. With the lack of studies with higher evidence level, a systematic review of highest quality is currently not feasible.

### Publication bias

With a previous understanding that tendinopathy is a degenerative disease with an absence of inflammation, it is possible that studies showing an inconsistent result will more likely be published. However, due to the great heterogenicity of the studies included in this review, plotting a funnel plot could not effectively identify the presence or absence of existing publication bias. Only publications in databases have been covered by our study, therefore the coverage of “grey literature” has not been evaluated [68].

### Signs of inflammation were present in the majority of tendinopathic tendons

According to our search results, signs of inflammation, including either the presence of inflammatory cells or an increase in inflammatory markers, were observed in 39 out of 53 studies. One common feature of the studies which reported an absence of inflammation was that the only method used was to identify inflammatory cells with H&E staining.

Considering the comparable clinical presentation, sampling method and staining method shared across the studies, we highly suspect the diagnosing accuracy of using H&E staining in the identification of inflammatory cells. As mentioned in a previous study [13], though there was an increase in macrophages present in chronic tendinopathic tendons, tenocytes still make up the majority of cells within the tendon. As macrophages make up less than 10% of the total population, it can be difficult to identify inflammatory cells in a crowded background of tenocytes. The previous thought that inflammation is absent in chronic tendinopathy could be a result of sub-optimal detection with an inappropriate method.

### Inflammatory cell types indicate chronic inflammation in tendinopathic tendons

Inflammatory cells observed in chronic tendinopathy were macrophages, lymphocytes, mast cells, and in some rare occasions, granulocytes. Except for granulocytes, the cell types indicate the state of inflammation as a chronic inflammation.

Macrophages are well known for its role in phagocytosis of infectious organisms [69]. However, the contribution of this cell type extends to many other systems including bone remodelling, erythropoiesis, brain and lung development [69]. It was also reported that macrophages play an important role in the regulation of inflammation [70]. Resident macrophages initiate the inflammatory response towards injury by recognizing damage associated molecular patterns (DAMPs) [69]. The process is followed by secretion of cytokines and eicosanoids, resulting in recruitment of inflammatory cells, with neutrophils being the first to enter the site [69]. The resolution of inflammation is also closely related to macrophage activity. The change in phenotype from M1 to a M2-like phenotype macrophage leads to a phenomenon known as the lipid mediator class switch [70].

Mast cells could have a significant role in tissue remodelling too. As reported in a previous review on mast cell physiology, it was reported that mast cell deficient mice initially had intact hair growth and bone density. However, defects were observed in case of injury, and tissue remodelling of the hair follicles and bone tissues were not comparable to that of healthy samples [71]. In another animal model on tendon injury, it was found that there were an increased expression of mast cells and myofibroblasts during the tendon healing process [72].

Lymphocytes are also known to be present in many autoimmune, inflammatory diseases such as Hashimoto thyroiditis and psoriasis [73]. It was hypothesized that the exaggerated recruitment of these cell types lead to un-controlled activation of macrophages, leading to excessive damage in cells and architecture [73]. It is possible that the same mechanism applies to tendons, where excessive lymphocyte activity could damage the extracellular matrices.

Granulocytes including neutrophils and eosinophils were reported in rare occasions. As mentioned above, neutrophils are the first cells to be recruited upon macrophage activation [69]. The presence of this cell type is indicative of an acute inflammatory state, reported only in cases of rupture.

### Inflammatory markers show an inconclusive pattern with current information

Unlike the information provided by the presence of inflammatory cells, current evidence on the inflammation markers shown in tendinopathic tendons do not show a consistent picture. As summarized in a review in 1997 [74], inflammatory mediators contribute to inflammation via complex pathways, but common mediators can be categorized to be present in acute inflammation, chronic inflammation, or both. In this review, the inflammatory mediators reported were a mixture of mediators in the acute, chronic and common group. It is also notable that inflammatory markers assessed vary greatly between studies. It is therefore very difficult to deduce the character of inflammation in chronic tendinopathy with the current information on detectable inflammatory markers.

However, study of the inflammatory markers in tendinopathic patients could potentially be of high importance. As mentioned in the limitations, the sampling of tendinopathic tendons in different stages could be challenging due to the invasive nature of tendon sampling. However, with increasing evidence that tendinopathy is associated with chronic inflammation, there may be systemic changes detectable as inflammatory markers in more assessible specimens, such as blood cells. Investigation on the systemic changes in tendinopathic patients could be a rewarding field of study.

### Possible explanations to varying presentation of inflammation between tendinopathic tendons

Causes of inflammation in tendinopathic tendons is currently unclear, however, 19/53 studies mentioned additional description to the recruited tendinopathic population, which may provide insights on whether the presentation of inflammation maybe limited to certain sub-groups. From our observations, inflammation is a character that often occur in chronic tendinopathy, regardless of location and chronicity. The presence of inflammation was also independent with previous treatments including steroid injection and NSAIDs. It was also directly mentioned in one included study that there were no association between steroid injections and the presentation of inflammation [20]. The previous understanding that chronic tendinopathy is an inflammation free disease may be due to low sensitivity of classical staining techniques using H&E. However, attention is drawn to two studies which reported a mere 20% rate of the presence of inflammatory cells in tendinopathic tendons despite adapting IHC for detection of related CD markers [27, 28]. The pattern is yet to be explained regarding the varying presentation of inflammation between tendinopathic tendons with similar clinical presentation.

One cause of inconsistency could be the existence of different stages to tendon injury. In some reports of acute tendon rupture, neutrophils and other granulocytes were reported present, likely acting as an acute response to trauma. However, current literature could not facilitate an educated deduction of factors associated with the presentation of inflammation. The cause, stage, and conservative managements received to treat tendinopathy could be some factors associated with the presentation of inflammation. Future studies with high quality are necessary to identify these variables.

The involvement of bacteria was also mentioned in some cases of tendinopathy. Although rarely assessed, there is a possibility that bacterial infection may play an important role in the presentation of chronic inflammation. Three studies which identified the presence of bacteria were excluded due to evidence level lower than 3. The Bacterial species identified in these studies were *Mycobacterium tuberculosis* [75, 76] and Borrelia [77]. One of the included studies identified staphylococcus genus as a possible initiating factor of inflammation [41]. In this study, the blood samples from the patients were negative for bacteria contrary to the presence of bacteria in the tendons. This suggests that the presence of bacteria was local to the AT. However, it is important to emphasize that the tendinopathic changes could favour the presence of bacteria and consequently the presence of bacteria in tendinopathic tissue may be secondary, as a result of the favouring environment. Further investigation into this topic may be a rewarding frontier.

The involvement of metabolic diseases associated with tendinopathy was also mentioned in several excluded case reports [78, 79]. The significance of such underlying disorders may also contribute to the varying presentation of inflammation observed. This concept is supported by previous reports that metabolic disorders including diabetes [80], obesity [81], gout [82], and hypothyroidism [83] can increase the risk of the development of tendinopathy. A systematic review in 2016 showed that various hormone receptors in tenocytes can be affected by hormonal imbalances of insulin, estrogens, thyroid hormones and growth hormone [84]. Hormone profile greatly impacts the inflammatory pathway, and one of such impacts is illustrated in a review describing inflammatory changes in diabetic and obese patients. Migration of inflammatory cells to adipose tissues lead to decreased availability and less effective healing in the tendon. Glycation of collagen and impaired cross linkage also contribute to sub-optimal healing [85]. As metabolic disorders impact on the hormonal profile differently, it is expected that inflammation following the metabolic insults may also vary.

### Consistency and inconsistency with existing literature

According to an editorial in 1998 [7], the most prominent lesion in chronic tendinopathies is a degenerative process with an absence of inflammatory cells [7]. It was acknowledged that histological findings were inconsistent, and that signs of inflammation were present in some of the tendon specimens. The explanation towards this phenomenon was that inflammation could be the “primum movens” of the development of tendinopathy. It was believed that the transient inflammatory state would ultimately lead to the typical presentation of tendinosis, with an absence of inflammation [7]. In simplified terms, it was suggested in this study that active inflammation, if any, should only occur at early stages of the development of tendinopathy. Search results from this systematic review do not fully agree with the classical study. Presence of inflammatory cells were reported in chronic tendinopathy in the included studies.

It is acknowledged that the current review is not the first to discuss on the presence of inflammation in chronic tendinopathy. A recent systematic review published in 2016 [86] also reached a similar conclusion. Quoting from the review, “the absence of inflammation in tendinopathy were more based on belief rather than scientific data”. The current review agrees with the idea that inflammation may be present, but the findings suggest that its presentation may not be as straightforward. By performing a more sophisticated search with more included, relevant studies, we were able to more subjectively assess the inconsistent presentation of inflammation in chronic tendinopathy between studies. Chronic inflammation is present in the majority of chronic tendinopathy using specific staining techniques, but there are exceptions which do not show positive staining of any inflammatory cells.

### Clinical significance

Tendinopathy is previously understood as a degenerative disorder with an absence of inflammation [37]. According to this review, it is likely that a chronic inflammation can be present in tendinopathic tendons. Therefore, the anti-inflammatory approach may well be supported in the conservative management of tendinopathy.

However, this does not mean that the current management strategy is flawless. Publications on the efficacy of current management strategies on tendinopathy have also shown that anti-inflammatory treatments like NSAIDs or corticosteroids only provide a short term relief of symptoms, and may have a negative impact on its structural healing [87, 88]. This result is consistent to the current finding since anti-inflammatory drugs suppress the inflammatory state to achieve pain relief. However, the effects do not last since chronic inflammation can be caused by the presence of a persistent stimulus, or a deranged cellular function to resolve inflammation.

The inhibition of the inflammatory process may also negatively impact the natural healing process of tendon healing, leading to further degenerative changes. This concept is supported by previous literature on the balance of metalloproteinases (MMPs) and its significance in maintaining healthy tendon homeostasis. MMP levels are regulated by inflammation [89], and it was mentioned that balanced MMP activity play an important role in maintaining structural integrity of tendons through constant extra-cellular matrix (ECM) remodelling [90, 91]. Inhibition to inflammation may in turns inhibit tendon homeostasis, leading to poor integrity in long term.

The cause leading to chronic inflammation could thus be multifactorial and varying among tendinopathic patients. However, rather than straightforward inhibition to the inflammatory process with NSAIDs or glucocorticoids, identification of factors leading to chronic inflammation, and corresponding targeted treatment could be the key in solving the burden caused by this common but chronic disease.

### Future studies

Current literature is insufficient to deduce a common pattern in regard to the presence of inflammation in chronic tendinopathy. To improve our understanding on this issue, more high quality studies with a large sample size and comparable detection methods is required. Known confounding factors such as activity level, previous history of anti-inflammatory treatment, and the chronicity of the tendon disorder must also be consistently documented in future for valuable comparison.

Also, it is acknowledged that the sampling of tendon samples with different stages of tendinopathy and confounding factors such as metabolic disease could be challenging. A variety of potentially triggering factors of inflammation in tendinopathy have been shown in this study, such as overuse, associated inflammatory and metabolic diseases, and bacterial involvement.

## Conclusion

This review suggest that inflammatory cells are observed in a proportion of tendinopathic tendons but not in all. Further controlled studies using comparable methods and sufficient sample sizes for various phases of tendon symptomatology are needed to allow any firm conclusion in regard to a potential common presentation of inflammation, and common pathway for the development of Tendinopathy.

## Data Availability

The datasets used and/or analysed during the current study are available from the corresponding author on reasonable request.

## Abbreviations

CASP: Critical Appraisal Skill Program
CD: Cluster of differentiation
CEBM: Oxford Centre of Evidence based Medicine
COX: Cyclooxygenase
DAMP: Damage associated molecular pattern
FGF: Fibroblast growth factor
H&E: Hematoxylin and eosin (stain)
IHC: Immunohistochemistry
IL: Interleukin
MRI: Magnetic Resonance Imaging
NSAID: Non-steroidal anti-inflammatory drug
PDGF: Platelet derived growth factor
PRISMA: Preferred Reporting Items for Systematic reviews and Meta-Analyses
TGFb: Transforming growth factor beta
